# Supplementary material for: Exploration of inositol 1,4,5-trisphosphate (IP3) regulated dynamics of N-terminal domain of IP3 receptor reveals early phase molecular events during receptor activation
Source: Sci Rep. 2019 Feb 21;9:2454. doi: 10.1038/s41598-019-39301-3 (PMC6385359; doi:10.1038/s41598-019-39301-3)
Supplement: Supplementary file 1 — Supplementary information [file 41598_2019_39301_MOESM1_ESM.docx]

**Supporting Information**

**Exploration of inositol 1, 4, 5-trisphosphate (IP_3_) regulated dynamics of N-terminal domain of IP_3_ receptor reveals early phase molecular events during receptor activation**

**Aneesh Chandran^1#*^, Xavier Chee^1^, David L. Prole^1^, Taufiq Rahman**^1*^

From the ^1^Department of Pharmacology, University of Cambridge, Tennis Court Road, Cambridge, UK CB2 1PD

^#^Present address: Molecular Biophysics Unit, Indian Institute of Science, Bangalore 560 012, India

*Correspondence:

Aneesh Chandran, Molecular Biophysics Unit, Indian Institute of Science, Bangalore 560 012, India, aneeshc@iisc.ac.in, Tel.:91-80-2293-2611; Taufiq Rahman, Department of Pharmacology, University of Cambridge, Tennis Court Road, Cambridge, UK CB2 1PD, mtur2@cam.ac.uk, 44-12233-34068.

**Table of Contents:**

1. Supporting Figures: Figures S1 – S10.
2. Movie S1 – S5


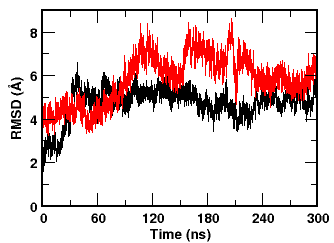


**(a)**


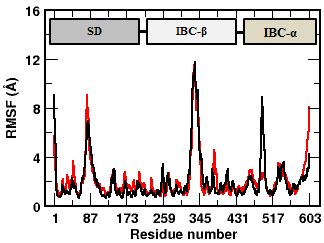


**(b)**

**Figure S1. The backbone structural deviations and residue-level fluctuations of apo and IP_3_-bound IP_3_R NT observed through MD simulations.** (a) The backbone RMSDs of simulated *apo* and IP_3_-bound IP_3_R NT from its minimized starting structure as a function of simulation time. (b) Residue-level RMSFs of IP_3_R NT in *apo* and IP_3_-bound state. The translational and rotational motions were removed by superimposing each snapshot prior to RMSD calculation. Color scheme: *apo*, black and IP_3_-bound, red.


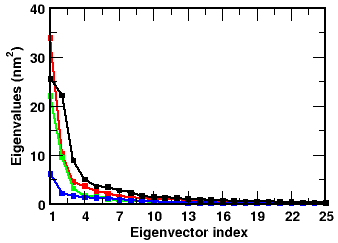


**Figure S2.** **Eigenvalue profile of essential motions analysed in different simulated systems.** Eigenvalues of the first 25 eigenvectors derived from PCA of the simulation trajectories of the different *apo* and IP_3_-bound IP_3_R NT systems. Color scheme: *apo* NT, black; IP_3_-bound NT, red; *apo* IBC, green; and IP_3_-bound IBC, blue.


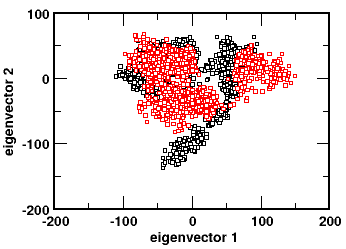


**(a)**


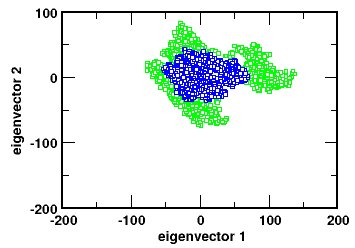


**(b)**

**Figure S3.** **Comparative sampling of essential motions in different systems simulated**. Two-dimensional projection of the simulated structures on the plane constituted by the first two principal components. (a) NT and (b) IBC. The color scheme is the same as in Figure S2.


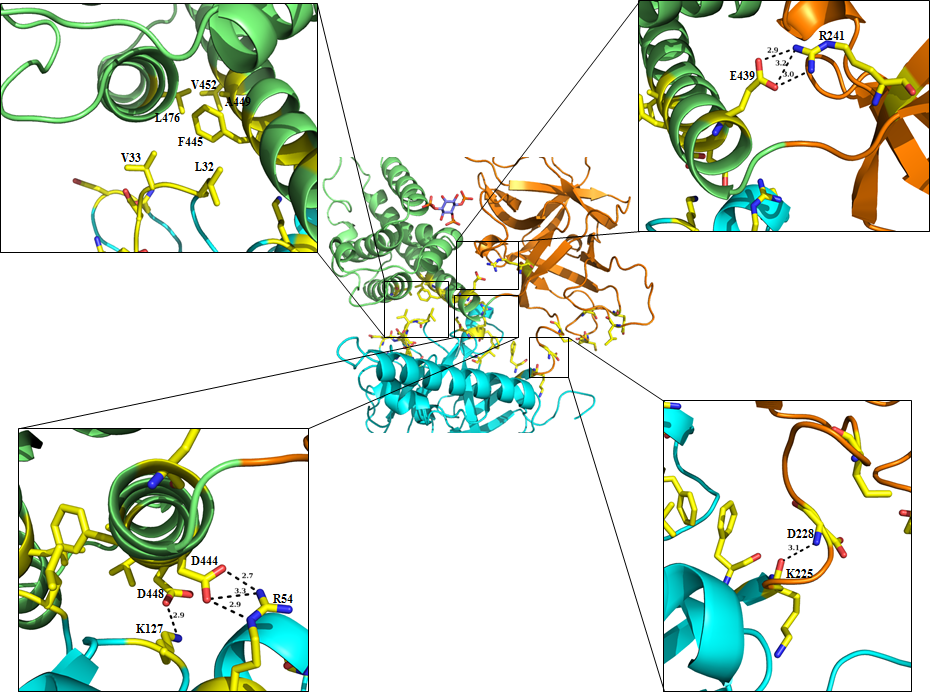


**(d)**

**(c)**

**(b)**

**(a)**

**Figure S4. Residue contacts at the SD – IBC interface in *apo* and IP_3_-bound NT**. (a) Hydrophobic interactions involving L32, V33, F445, A449, V452, and L476 at the α-interface. (b) R241 – E439 salt bridge between IBC-β and IBC-α domains. (c) Ionic contacts between SD and IBC-α involve R54 – D444 and K127 – D448 interactions. (d) As observed from the crystal structures (PDB ID: 3UJ0 and 3UJ4), the K225 – D228 salt bridge is formed within the connecting turn between the SD and IBC-β.


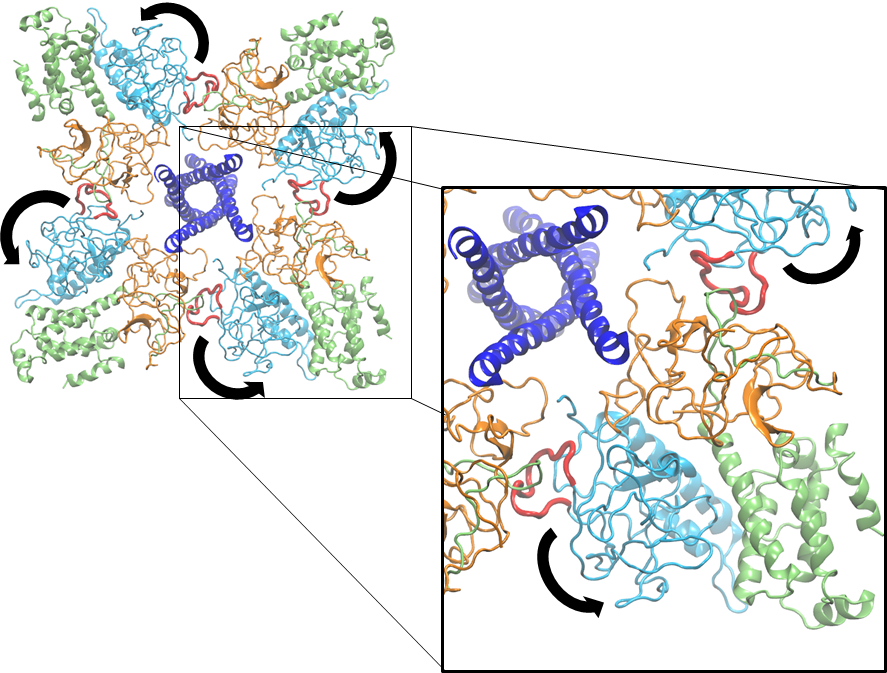


**Figure S5. Structural arrangement of monomeric NT units in a functional tetrameric form of IP_3_R NT (PDB ID: 3JAV).** The color scheme is the same as in Figure 1. The monomeric units are arranged in such a way that the SD (cyan) of one monomer interacts with the IBC-β of the neighboring monomer through the HS loop (red) of SD. The IP_3_ binding causes the SD to twist towards IBC (shown in curved arrow), disrupting the HS loop – IBC-β interaction at the inter-monomeric interface.

**
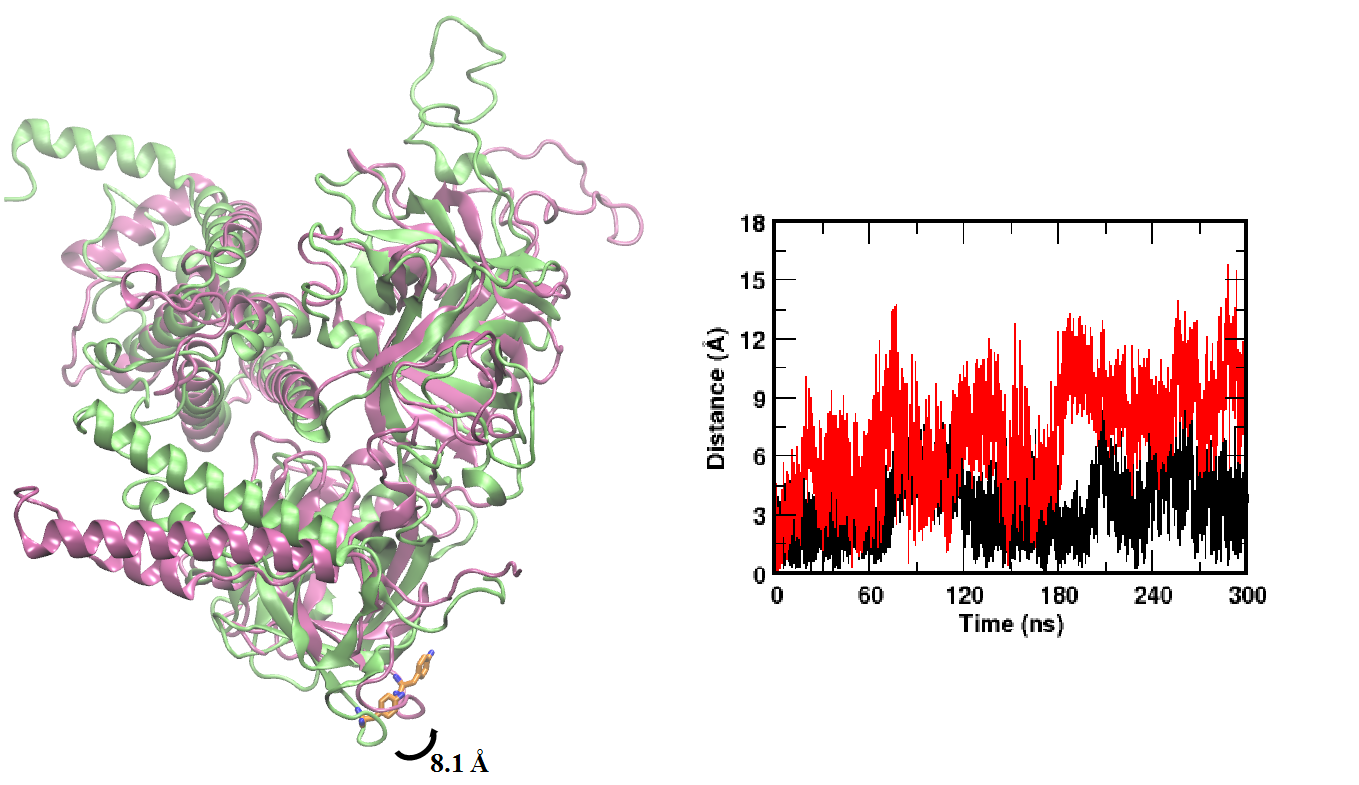
**

**(b)**

**(a)**

**Figure S6. The extent of translational displacement of HS loop during twist motion of the SD.** (a) Superposition of ensemble-averaged apo (green) and IP_3_-bound (pink) NT by overlaying the IBC domain shows an average displacement of Tyr167 C_α_ by 8.1 Å. (b) A plot of Tyr167 C_α_ distance from its minimized initial position as a function of simulation time. Color scheme: *apo* NT, black; and IP_3_-bound NT, red.


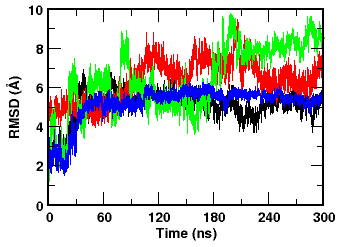


**Figure S7. The backbone RMSDs of the IBC domain in each of the simulated systems (see Table 1) from its minimized starting structure as a function of simulation time**. The translational and rotational motions were removed by superimposing each snapshot prior to RMSD calculation. The color scheme is the same as in Figure S2.


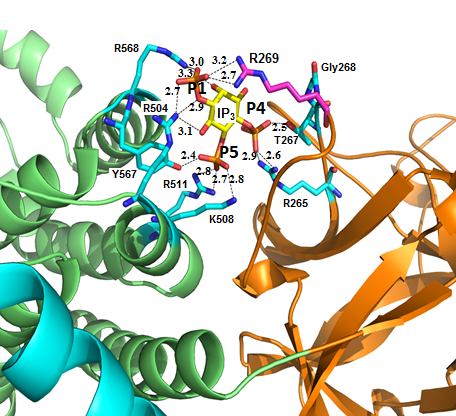


**(a)**


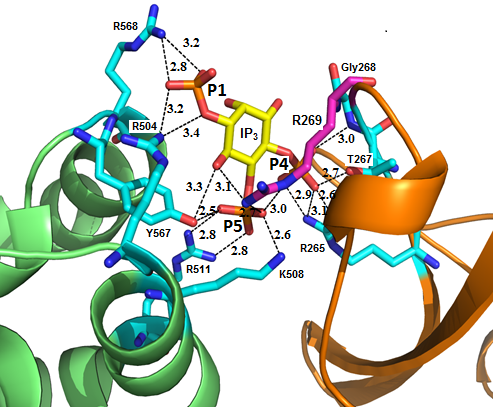


**(b)**

**Figure S8. Average structure of IP_3_-bound (a) NT and (b) IBC, showing the interaction pattern of IP_3_ with the protein.** For clarity, only the IP_3_ and adjacent protein residues are shown in stick representation. Possible modes of interactions are indicated by dotted lines with their average distances. Color scheme: O, red; N, blue; protein C, cyan; IP_3_ C, yellow; IP_3_ S, orange; and Arg269 C, magenta. Hydrogens are omitted for clarity.

**
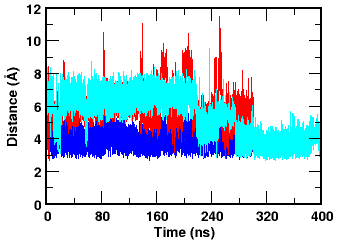
**

**Figure S9. Arg269 makes additional interaction with IP_3_ in the absence of the SD.** Time evolution of Arg269 guanidinium NH_2_ – C6'-OH distance during the simulation of different systems. Color scheme: IP_3_-bound NT, red; IP_3_-bound IBC, blue; and SD-knockout (system 5), cyan. It is evident from the figure that the flipping of Arg269 (~ at 280 ns) in the absence of the SD in system 5 helps the guanidinum group to make additional h-bond with C6'-OH of the bound IP_3_.

**R54**

DmIP3R MGDNIIGSASFLHLGDIVSLYAEGSVCGFLSTLGLVDDRTVVCPEAGDLSCPPKKF**R**DCL 60

rIP3R1 ---MSDKMSSFLHIGDICSLYAEGSTNGFISTLGLVDDRCVVQPEAGDLNNPPKKF**R**DCL 57

mIP3R1 ---MSDKMSSFLHIGDICSLYAEGSTNGFISTLGLVDDRCVVQPEAGDLNNPPKKF**R**DCL 57

hIP3R1 ---MSDKMSSFLHIGDICSLYAEGSTNGFISTLGLVDDRCVVQPETGDLNNPPKKF**R**DCL 57

hIP3R3 ----MSEMSSFLHIGDIVSLYAEGSVNGFISTLGLVDDRCVVEPAAGDLDNPPKKF**R**DCL 56

rIP3R3 ----MNEMSSFLHIGDIVSLYAEGSVNGFISTLGLVDDRCVVEPAAGDLDNPPKKF**R**DCL 56

mIP3R3 ----MNEMSSFLHIGDIVSLYAEGSVNGFISTLGLVDDRCVVEPAAGDLDNPPKKF**R**DCL 56

hIP3R2 ---MTEKMSSFLYIGDIVSLYAEGSVNGFISTLGLVDDRCVVHPEAGDLANPPKKF**R**DCL 57

rIP3R2 ---MSDKMSSFLYIGDIVSLYAEGSVNGFISTLGLVDDRCVVHPEAGDLTNPPKKF**R**DCL 57

mIP3R2 ---MSDKMSSFLYIGDIVSLYAEGSVNGFISTLGLVDDRCVVHPEAGDLANPPKKF**R**DCL 57

:***::*** *******. **:********* ** * :*** *********

DmIP3R IKICPMNRYSAQKQFWKAAK--QSASSNTDPNLLKRLHHAAEIEKKQNETENKKLLGTSI 118

rIP3R1 FKLCPMNRYSAQKQFWKAAK--PGANSTTDAVLLNKLHHAADLEKKQNETENRKLLGTVI 115

mIP3R1 FKLCPMNRYSAQKQFWKAAK--PGANSTTDAVLLNKLHHAADLEKKQNETENRKLLGTVI 115

hIP3R1 FKLCPMNRYSAQKQFWKAAK--PGANSTTDAVLLNKLHHAADLEKKQNETENRKLLGTVI 115

hIP3R3 FKVCPMNRYSAQKQYWKAKQTKQDKEKIADVVLLQKLQHAAQMEQKQNDTENKKVHGDVV 116

rIP3R3 FKVCPMNRYSAQKQYWKAKQTKQDKEKIADVVLLQKLQHAAQMEQKQNDTENKKVHGDVV 116

mIP3R3 FKVCPMNRYSAQKQYWKAKQTKQDKEKIADVVLLQKLQHAAQMEQKQNDTENKKVHGDVV 116

hIP3R2 FKVCPMNRYSAQKQYWKAKQAKQGNH--TEAALLKKLQHAAELEQKQNESENKKLLGEIV 115

rIP3R2 FKVCPMNRYSAQKQYWKAKQAKQGNH--TEAALLKKLQHAAELEQKQNESENRKLLGEIV 115

mIP3R2 FKVCPMNRYSAQKQYWKAKQAKQGNH--TEAALLKKLQHAAELEQKQNESENRKLLGEIV 115

:*:***********:*** : . :: **::*:***::*:***::**:*: * :

DmIP3R QYGRAVVQLLHLKSNKYLTVNKRLPSLLEKNAMRVYLDANGNEGSWFYIKPFYKLRSIGD 178

rIP3R1 QYGN-VIQLLHLKSNKYLTVNKRLPALLEKNAMRVTLDEAGNEGSWFYIQPFYKLRSIGD 174

mIP3R1 QYGN-VIQLLHLKSNKYLTVNKRLPALLEKNAMRVTLDEAGNEGSWFYIQPFYKLRSIGD 174

hIP3R1 QYGN-VIQLLHLKSNKYLTVNKRLPALLEKNAMRVTLDEAGNEGSWFYIQPFYKLRSIGD 174

hIP3R3 KYGS-VIQLLHMKSNKYLTVNKRLPALLEKNAMRVTLDATGNEGSWLFIQPFWKLRSNGD 175

rIP3R3 KYGS-VIQLLHMKSNKYLTVNKRLPALLEKNAMRVTLDATGNEGSWLFIQPFWKLRSNGD 175

mIP3R3 KYGS-VIQLLHMKSNKYLTVNKRLPALLEKNAMRVTLDATGNEGSWLFIQPFWKLRSNGD 175

hIP3R2 KYSN-VIQLLHIKSNKYLTVNKRLPALLEKNAMRVSLDAAGNEGSWFYIHPFWKLRSEGD 174

rIP3R2 KYSK-VIQLLHIKSNKYLTVNKRLPALLEKNAMRVSLDAAGNEGSWFYIHPFWKLRSEGD 174

mIP3R2 KYSN-VIQLLHIKSNKYLTVNKRLPALLEKNAMRVSLDAAGNEGSWFYIHPFWKLRSEGD 174

:*. *:****:*************:********* ** ******::*:**:**** **

**Figure S10.** **Multiple sequence alignment of the N terminus of different IP_3_R isoforms from rat, mouse, human and Drosophila.** Clustal Omega (https://www.ebi.ac.uk/Tools/msa/clustalo/) in its default mode was used to align amino acid sequences of rat IP_3_R1 (rIP_3_R1, uniprot ID: P29994), rat IP_3_R2 (rIP_3_R2, uniprot ID: P29995), rat IP_3_R3 (rIP3R3, uniprot ID: Q63269), mouse IP_3_R1 (mIP_3_R1, uniprot ID: P11881), mouse IP_3_R2 (mIP_3_R2, uniprot ID: Q9Z329), mouse IP_3_R3 (mIP_3_R3, uniprot ID: P70227), human IP_3_R1 (hIP_3_R1, uniprot ID: Q14643), human IP_3_R2 (hIP_3_R2, uniprot ID: Q14571), human IP_3_R3 (hIP_3_R3, uniprot ID: Q14573) and *Drosophila melanogaster* IP_3_R (DmIP_3_R1, uniprot ID: P29993). Arg54, Arg269, Glu283 and Asp444 are highlighted.

**Figure S10 contd.**

DmIP3R YVVVVGDKVILSPVNADQQNLHVAANYELPDNPGCKEVNVLNSSTSWKISLFMEHKENQE 238

rIP3R1 SV-VIGDKVVLNPVNAGQPL--HASSHQLVDNPGCNEVNSVNCNTSWKIVLFMKWSDNKD 231

mIP3R1 SV-VIGDKVVLNPVNAGQPL--HASSHQLVDNPGCNEVNSVNCNTSWKIVLFMKWSDNKD 231

hIP3R1 SV-VIGDKVVLNPVNAGQPL--HASSHQLVDNPGCNEVNSVNCNTSWKIVLFMKWSDNKD 231

hIP3R3 NV-VVGDKVILNPVNAGQPL--HASNYELSDNAGCKEVNSVNCNTSWKINLFMQFRDHLE 232

rIP3R3 NV-VVGDKVILNPVNAGQPL--HASNYELSDNVGCKEVNSVNCNTSWKINLFMQFRDHLE 232

mIP3R3 NV-VVGDKVILNPVNAGQPL--HASNYELSDNAGCKEVNSVNCNTSWKINLFMQFRDHLE 232

hIP3R2 NI-VVGDKVVLMPVNAGQPL--HASNIELLDNPGCKEVNAVNCNTSWKITLFMKYSSYRE 231

rIP3R2 NI-VVGDKVVLMPVNAGQPL--HASNVELLDNPGCKEVNAVNCNTSWKITLFMKFSSYRE 231

mIP3R2 NI-VVGDKVVLMPVNAGQPL--HASNVELLDNPGCKEVNAVNCNTSWKITLFMKFSSYRE 231

: *:****:* ****.* *:. :* ** **:*** :*..***** ***: . :

**R269**  **E283**

DmIP3R HILKGGDVVRLFHAEQEKFLTMDEYKKQYHVFLRTTG**R**TSATAATSSKALW**E**IEVVQHDS 298

rIP3R1 DILKGGDVVRLFHAEQEKFLTCDEHRKKQHVFLRTTG**R**QSATSATSSKALW**E**VEVVQHDP 291

mIP3R1 DILKGGDVVRLFHAEQEKFLTCDEHRKKQHVFLRTTG**R**QSATSATSSKALW**E**VEVVQHDP 291

hIP3R1 DILKGGDVVRLFHAEQEKFLTCDEHRKKQHVFLRTTGRQSATSATSSKALW**E**VEVVQHDP 291

hIP3R3 EVLKGGDVVRLFHAEQEKFLTCDEYKGKLQVFLRTTLRQSATSATSSNALW**E**VEVVHHDP 292

rIP3R3 EVLKGGDVVRLFHAEQEKFLTCDEYRGKLQVFLRTTLRQSATSATSSNALW**E**VEVVHHDP 292

mIP3R3 EVLKGGDVVRLFHAEQEKFLTCDEYRGKLQVFLRTTLRQSATSATSSNALW**E**VEVVHHDP 292

hIP3R2 DVLKGGDVVRLFHAEQEKFLTCDEYEKKQHIFLRTTLRQSATSATSSKALW**E**IEVVHHDP 291

rIP3R2 DVLKGGDVVRLFHAEQEKFLTCDDYEKKQHIFLRTTLRQSATSATSSKALW**E**IEVVHHDP 291

mIP3R2 DVLKGGDVVRLFHAEQEKFLTCDDYEKKQHIFLRTTLRQSATSATSSKALW**E**IEVVHHDP 291

.:******************* *::. : ::***** * ***:****:****:***:**

DmIP3R CRGGAGDWNSLYRFKHLATGHYLAAEAEIDVSAGAMSATSASGHDLHLGDCSKDSGLSCS 358

rIP3R1 CRGGAGYWNSLFRFKHLATGHYLAAEVDPDFEEECLEFQPSV-----------DPDQD-- 338

mIP3R1 CRGGAGYWNSLFRFKHLATGHYLAAEVDPDFEEECLEFQPSV-----------DPDQD-- 338

hIP3R1 CRGGAGYWNSLFRFKHLATGHYLAAEVDPDFEEECLEFQPSV-----------DPDQD-- 338

hIP3R3 CRGGAGHWNGLYRFKHLATGNYLAAEENPSYKGDASDPKAAG------------MGAQ-- 338

rIP3R3 CRGGAGHWNGLYRFKHLATGNYLAAEENPSYKGDVSDPKAAG------------PGAQ-- 338

mIP3R3 CRGGAGHWNGLYRFKHLATGNYLAAEENPSYKGDVSDPKAAG------------LGAQ-- 338

hIP3R2 CRGGAGQWNSLFRFKHLATGNYLAAELNPDYRDAQNEGKNVR------------DGVP-- 337

rIP3R2 CRGGAGQWNSLFRFKHLATGNYLAAELNPDYRDAQNEGKTVR------------DGEL-- 337

mIP3R2 CRGGAGQWNSLFRFKHLATGNYLAAELNPDYRDAQNEGKNVK------------DGEI-- 337

****** **.*:********:***** : . . .

DmIP3R TMNSTINDKPKGKQYRLVSVPYSADIASVFVLDATTMARPDSLVPQSSYVRLQHICSNTW 418

rIP3R1 ASRSRLRNAQEKMVYSLVSVPEGNDISSIFELDPTTLRGGDSLVPRNSYVRLRHLCTNTW 398

mIP3R1 ASRSRLRNAQEKMVYSLVSVPEGNDISSIFELDPTTLRGGDSLVPRNSYVRLRHLCTNTW 398

hIP3R1 ASRSRLRNAQEKMVYSLVSVPEGNDISSIFELDPTTLRGGDSLVPRNSYVRLRHLCTNTW 398

hIP3R3 -GRTGRRNAGEKIKYCLVAVPHGNDIASLFELDPTTLQKTDSFVPRNSYVRLRHLCTNTW 397

rIP3R3 -SRTGRRNAGEKIKYRLVAVPHGNDIASLFELDPTTLQKTDSFVPRNSYVRLRHLCTNTW 397

mIP3R3 -GRTGRRNAGEKIKYRLVAVPHGNDIASLFELDPTTLQKTDSFVPRNSYVRLRHLCTNTW 397

hIP3R2 PTSKKKRQAGEKIMYTLVSVPHGNDIASLFELDATTLQRADCLVPRNSYVRLRHLCTNTW 397

rIP3R2 PTSKKKHQAGEKIMYTLVSVPHGNDIASLFELDATTLQRADCLVPRNSYVRLRHLCTNTW 397

mIP3R2 PTPKKKRQAGEKIMYTLVSVPHGNDIASLFELDATTLQRADCLVPRNSYVRLRHLCTNTW 397

. .: : * **:** . **:*:* ** **: *.:**:.*****:*:*:***

**Figure S10 contd.**

**D444**

DmIP3R VHATSIPIDADDDKPVMSMVCCSPIKEDKEAFALIPVSPVEVRDL**D**FANDACKVLATVTS 478

rIP3R1 VHSTNIPIDKEEEKPVMLKIGTSPLKEDKEAFAIVPVSPAEVRDL**D**FANDASKVLGSIAG 458

mIP3R1 VHSTNIPIDKEEEKPVMLKIGTSPLKEDKEAFAIVPVSPAEVRDL**D**FANDASKVLGSIAG 458

hIP3R1 VHSTNIPIDKEEEKPVMLKIGTSPVKEDKEAFAIVPVSPAEVRDL**D**FANDASKVLGSIAG 458

hIP3R3 IQSTNVPIDIEEERPIRLMLGTCPTKEDKEAFAIVSVPVSEIRDL**D**FANDASSMLASAVE 457

rIP3R3 IQSTNAPIDVEEERPIRLMLGTCPTKEDKEAFAIVSVPVSEIRDL**D**FANDASSMLASAVE 457

mIP3R3 IQSTNAPIDVEEERPIRLMLGTCPTKEDKEAFAIVSVPVSEIRDL**D**FANDASSMLASAVE 457

hIP3R2 VTSTSIPIDTDEERPVMLKIGTCQTKEDKEAFAIVSVPLSEVRDL**D**FANDANKVLATTVK 457

rIP3R2 VTSTSIPIDTEEERPVMLKIGTCQTKEDKEAFAIVCVPLSEVRDL**D**FANDANKVLATTVK 457

mIP3R2 VTSTTIPIDTEEERPVMLKIGTCQTKEDKEAFAIVCVPLSEVRDL**D**FANDANKVLATTVK 457

: :*. *** ::::*: : . ********:: * *:********* .:*.: .

DmIP3R KLDNGSISINERRALISLLQDIVYFIAGMENEQNKTKALEFTIKNPIRDRQKLLREQYIL 538

rIP3R1 KLEKGTITQNERRSVTKLLEDLVYFVTGGTNSG--QDVLEVVFSKPNRERQKLMREQNIL 516

mIP3R1 KLEKGTITQNERRSVTKLLEDLVYFVTGGTNSG--QDVLEVVFSKPNRERQKLMREQNIL 516

hIP3R1 KLEKGTITQNERRSVTKLLEDLVYFVTGGTNSG--QDVLEVVFSKPNRERQKLMREQNIL 516

hIP3R3 KLNEGFISQNDRRFVIQLLEDLVFFVSDVPNNG--QNVLDIMVTKPNRERQKLMREQNIL 515

rIP3R3 KLNEGFISQNDRRFVIQLLEDLVFFVSDVPNNG--QNVLDIMVTKPNRERQKLMRDENIL 515

mIP3R3 KLNEGFISQNDRRFVIQLLEDLVFFVSDVPNNG--QNVLDIMVTKPNRERQKLMREQNIL 515

hIP3R2 KLENGTITQNERRFVTKLLEDLIFFVADVPNNG--QEVLDVVITKPNRERQKLMREQNIL 515

rIP3R2 KLENGSITQNERRFVTKLLEDLIFFVADVTNNG--QDVLDVVITKPNRERQKLMREQNIL 515

mIP3R2 KLENGSITQNERRFVTKLLEDLIFFVADVTNNG--QDVLDVVITKPNRERQKLMREQNIL 515

**::* *: *:** : .**:*:::*::. *. ..*:. ..:* *:****:*:: **

DmIP3R KQLFKILQGPFQEHTAGDGPFLRLDELSDPKNSPYKNIFRLCYRILRLSQQDYRKNQEYI 598

rIP3R1 KQIFKLLQAPFTD--CGDGPMLRLEELGDQRHAPFRHICRLCYRVLRHSQQDYRKNQEYI 574

mIP3R1 KQIFKLLQAPFTD--CGDGPMLRLEELGDQRHAPFRHICRLCYRVLRHSQQDYRKNQEYI 574

hIP3R1 KQIFKLLQAPFTD--CGDGPMLRLEELGDQRHAPFRHICRLCYRVLRHSQQDYRKNQEYI 574

hIP3R3 KQVFGILKAPFRE-KGGEGPLVRLEELSDQKNAPYQHMFRLCYRVLRHSQEDYRKNQEHI 574

rIP3R3 KQIFGILKAPFRD-KGGEGPLVRLEELSDQKNAPYQYMFRLCYRVLRHSQEDYRKNQEHI 574

mIP3R3 KQIFGILKAPFRD-KGGEGPLVRLEELSDQKNAPYQYMFRLCYRVLRHSQEDYRKNQEHI 574

hIP3R2 AQVFGILKAPFKE-KAGEGSMLRLEDLGDQRYAPYKYMLRLCYRVLRHSQQDYRKNQEYI 574

rIP3R2 AQVFGILKAPFKE-KAGEGSMLRLEDLGDQRYAPYKYVLRLCYRVLRHSQQDYRKNQEYI 574

mIP3R2 AQVFGILKAPFKE-KAGEGSMLRLEDLGDQRYAPYKYVLRLCYRVLRHSQQDYRKNQEYI 574

*:* :*:.** : *:* ::**::*.* : :*:: : *****:** **:*******:*

DmIP3R AKHFGLMQKQIGYDILAEDTITALLHNNRK 628

rIP3R1 AKQFGFMQKQIGYDVLAEDTITALLHNNRK 604

mIP3R1 AKQFGFMQKQIGYDVLAEDTITALLHNNRK 604

hIP3R1 AKQFGFMQKQIGYDVLAEDTITALLHNNRK 604

hIP3R3 AKQFGMMQSQIGYDILAEDTITALLHNNRK 604

rIP3R3 AKQFGMMQSQIGYDILAEDTITALLHNNRK 604

mIP3R3 AKQFGMMQSQIGYDILAEDTITALLHNNRK 604

hIP3R2 AKNFCVMQSQIGYDILAEDTITALLHNNRK 604

rIP3R2 AKNFCVMQSQIGYDILAEDTITALLHNNRK 604

mIP3R2 AKNFCVMQSQIGYDILAEDTITALLHNNRK 604

**:* .**.*****:***************

**Movie S1.** Dynamics of *apo* IP_3_R NT along the first principal component obtained from PCA.

**Movie S2.** Domain motions in IP_3_-bound IP_3_R NT along the first principal component obtained from PCA.

**Movie S3.** Bending motion of *apo* IP_3_R IBC domains along the first principal component obtained from PCA.

**Movie S4.** Dynamics of IP_3_-bound IP_3_R IBC along the first principal component obtained from PCA.

**Movie S5.** Flipping mechanism of Arg269 in the absence of the SD of IP_3_-bound IP_3_R NT
